# Pathogenic mechanisms of osteogenesis imperfecta, evidence for classification

**DOI:** 10.1186/s13023-023-02849-5

**Published:** 2023-08-09

**Authors:** Hongjie Yu, Changrong Li, Huixiao Wu, Weibo Xia, Yanzhou Wang, Jiajun Zhao, Chao Xu

**Affiliations:** 1 Department of Endocrinology, Shandong Provincial Hospital Affiliated to Shandong First Medical University, Jinan, Shandong 250021 China; 2 Shandong Institute of Endocrine and Metabolic Diseases, Jinan, Shandong 250021 China; 3 Shandong Engineering Research Center of Stem Cell and Gene Therapy for Endocrine and Metabolic Diseases, Jinan, Shandong 250021 China; 4 Department of Endocrinology, Key Laboratory of Endocrinology, Peking Union Medical College Hospital, National Commission of Health, Chinese Academy of Medical Sciences and Peking Union Medical College, Beijing, China 100730; 5 Department of Pediatric Orthopedics, Shandong Provincial Hospital Affiliated to Shandong First Medical University, Jinan, Shandong 250021 China

**Keywords:** Osteogenesis imperfecta, Pathogenic mechanism, Classification

## Abstract

Osteogenesis imperfecta (OI) is a connective tissue disorder affecting the skeleton and other organs, which has multiple genetic patterns, numerous causative genes, and complex pathogenic mechanisms. The previous classifications lack structure and scientific basis and have poor applicability. In this paper, we summarize and sort out the pathogenic mechanisms of OI, and analyze the molecular pathogenic mechanisms of OI from the perspectives of type I collagen defects(synthesis defects, processing defects, post-translational modification defects, folding and cross-linking defects), bone mineralization disorders, osteoblast differentiation and functional defects respectively, and also generalize several new untyped OI-causing genes and their pathogenic mechanisms, intending to provide the evidence of classification and a scientific basis for the precise diagnosis and treatment of OI.

## Introduction

Osteogenesis imperfecta, also known as brittle bone disease, is a rare genetic heterogeneous connective tissue disorder with an incidence of 1 in 15,000 to 20,000 newborns. The skeletal phenotype of patients with OI is characterized by reduced bone density, increased bone fragility, recurrent fractures, and progressive skeletal deformities. More common extra skeletal phenotypes include blue sclera, dentinogenesis imperfecta, and hearing impairment. In the past, OI was thought to be caused only by dominant mutations in the genes encoding type I collagen (COL1A1 and COL1A2) resulting in defective type I collagen, and patients with OI were classified as Sillence types I-IV based on the clinical phenotype [1]. However, with the discovery of other rare causative genes, OI is now considered to be a disease “associated” with type I collagen. Rare causative genes are involved in post-translational modifications, processing, folding, and cross-linking of type I collagen, but also in bone mineralization and osteoblast differentiation. Since the pathogenic mechanism of each OI subtype is diverse, their clinical features present high heterogeneity.

In order to accurately classify OI, researchers have proposed genetic typing of OI based on the causative genes. OI has a wide variety of causative genes, complex pathogenesis, and typing, and its mode of inheritance covers autosomal dominant (AD), autosomal recessive (AR), and X-linked recessive (XR) inheritance (Table 1). However, the current classification of OI is randomly classified into a type whenever a pathogenic gene is identified. Therefore, it is highly necessary to consider the reclassification of OI according to molecular mechanisms and clinical features.

**Table 1:** Pathogenesis and genetic typing of OI

Typing	Pathogenic mechanism	Sub-type	Pre-existing typing	Pathogenic genes	Mutant proteins	Mode of inheritance	Phenotype lightness and weight
Type 1	Type I Collagen defects	1 A	Type I	COL1A1	α1 chain	AD	Mild
			Type II-IV	COL1A1, COL1A2	α2 chain	AD	More severe clinical phenotype or lethality
		1B	Type XIII	BMP1	BMP1	AR	More severe
			Type VIIC	ADAMT-2	ADAMT-2	AR	ESD, not OI
		1 C	Type VII	CRTAP	CRTAP	AR	Severe to lethality
			Type VIII	LEPRE1	P3H1	AR	Severe to lethality
			Type IX	PPIB	CypB	AR	Extremely rare
			Type XIV	TMEMB38	TRIC-B	AR	Asymptomatic individual to severe OI
		1D	Type X	SERPINH1	HSP47	AR	Severe
			Type XI	FKBP10	FKBP65	AR	
			-	KDELR2	KDELR2	AR	
			-/BS	PLOD2	LH2	AR	
Type 2	Bone mineralization defects		Type V	IFITM5	BRIL	AD	Usually a moderate OI, similar in severity to type IV OI
			Type VI	SERPINF1	PEDF	AR	Severe bone dysplasia
			Special Type VI	IFITM5	BRIL (Ser40Leu)	AD	More severe than typical Type VI OI
Type 3	Defective osteoblast differentiation and function		Type XVI	CREB3L1	OASIS	AR	Mild or lethality
			Type XVIII	MBTPS2	S2P	XR	Moderate severity
			Type XV	WNT1	WNT1	AR	Moderate to progressive deformation
			Type XII	SP7	Osterix	AR	
			Type XVII	SPRAC	Osteonectin	AR	
Type 4	Unclassified and untyped OI		-	FAM46A	FAM46A	AR	
			-	MESD	MESD	AR	Can cause stillbirth, drugs that enhance Wnt signaling such as anti-sclerostin antibodies may have potential therapeutic efficacy
			-	CCDC134	CCDC134	AR	

OI: osteogenesis imperfecta; BS: Bruck syndrome; EDS: Ehlers-Danlos syndrome; AD: autosomal dominant; AR: autosomal recessive; XR: X-linked recessive

In this paper, we review the phenotypic severity and pathogenic mechanisms and genetic typing of OI, and classify it into types 1–4 based on pathogenic mechanisms to provide the evidence of classification.

## Type 1: type I collagen defects and mechanisms

### Type 1A: type I collagen synthesis defect (Sillence type I-IV OI)

Type I collagen, the most important component of the bone extracellular matrix, is a heterotrimer consisting of two α1 chains encoded by the COL1A1 gene and a helix of α2 chains encoded by the COL1A2 gene. Over 80% of OIs are caused by dominant mutations in the COL1A1 and COL1A2 genes resulting in defects in the amount or structure of type I collagen. In general, nonsense mutations, shift mutations, and splice mutations in the COL1A1 gene result in reduced amounts of type I collagen and are associated with mild OI, corresponding to type I of the Sillence typing. However, missense mutations in the COL1A1 and COL1A2 genes, especially glycine substitution in the type I collagen triple helix structure, destabilize the triple helix structure and delay type I collagen folding, causing a more severe clinical phenotype or lethality, corresponding to types II-IV in Sillence typing [2]. How different glycine substitutions lead to different clinical severity of mechanisms remains incompletely understood, and it is currently not possible to mechanistically predict the severity of clinical phenotypes resulting from glycine substitution.

### Type 1B: type I collagen processing defects (Sillence type XIII OI, EDS)

The precursor of type I collagen, procollagen type I, is composed of a triple helix structure with a globular amino-terminal and carboxy-terminal propeptide at both ends, in which the carboxy-terminal propeptide plays an important role in the mutual recognition and binding of collagen chains [3]. Both premature termination codon (PTC) appearance and amino acid substitution mutations have been reported in carboxy-terminal prepropeptides, in which when PTC leads to nonsense-mediated mRNA decay (NMD), mutant collagen chain synthesis is reduced, and the amount of type I collagen is resulting in a milder phenotype.

However, failure of the NMD mechanism to recognize PTC can lead to the formation of proto-type I collagen involving mutant collagen chains, which can affect the folding and over-modification of procollagen type I, leading to severe or fatal phenotypes [4]. Missense mutations affecting the procollagen C-propeptide cleavage site are associated with a highly distinct OI phenotype of mild to moderate severity characterized by high bone mass. The data of Rolvien T et al. independently demonstrate the presence of an abnormal phenotype of high bone density OI due to the inhibition of precollagen C-peptide cleavage. Although a high bone mass OI phenotype has been previously reported in children, their results suggest that high bone mass persists into late adulthood [5]. In addition, disulfide bonds between collagen chains are important for the initial phase of type I collagen assembly, and mutations in the carboxy-terminal prepropeptide that affect disulfide bonds within the chains delay the binding and secretion of the mutant collagen chains [6]. The intracellular retention of abnormal pre-I collagen further causes endoplasmic reticulum stress, which is associated with stimulation of autophagy, induction of apoptosis, and impaired osteoblast differentiation [7].

After normal pre-type I collagen is secreted extracellularly, its carboxy-terminal and amino-terminal propeptides are cleaved by bone morphogenetic protein 1 (BMP1) and a disintegrin and metalloproteinase with thrombospondin motifs 2 (ADAMT-2), respectively, to form mature type I collagen and further assemble into collagen fibers. Mutations in BMP1, the cleavage enzyme of the carboxy-terminal prepropeptide, cause abnormal processing of procollagen type I, resulting in the phenotypically more severe Sillence type XIII OI, but notably, Sillence type XIII OI exhibits increased bone mineralization [8]. The mechanism of its role in bone mineralization has recently been recognized and is being studied in mouse models. Interestingly, mutations in the metalloprotease ADAMTS-2 cause Ehlers-Danlos syndrome (type VIIA or VIIB) but not OI [9]. Mutations at the amino-terminal peptide cleavage site of pre-type I collagen also cause Ehlers-Danlos syndrome (type VIIC) but not OI [10].

### Type 1C: defective post-translational modification of type I collagen (Sillence type VII, VIII, IX, XIV OI)

The formation of pre-type I collagen is a very complex process that requires several post-translational modifications and folding. Prolyl 4-hydroxylase 1 (P4H1), lysyl hydroxylase 1 (LH1), and the proline hydroxylase complex all play important roles in the modification of the collagen chain. The proline hydroxylase complex consists of prolyl 3-hydroxylase 1 (P3H1), cartilage-associated protein (CRTAP), and cyclophilin B (CypB). Mutations in the gene encoding the proline hydroxylase complex, which is critical for proline 3-hydroxylation at position 986 on the specific α1 chain of procollagen type I and collagen chain folding, also further reveal a rare form of autosomal invisible genetic osteogenesis imperfecta. Mutations in the CRTAP gene lead to Sillence type VII OI and mutations in the LEPRE1 gene, which encodes the P3H1 protein, lead to Sillence type VIII OI, and both gene mutations result in delayed folding of the collagen chain and consequently in excessive hydroxylation of lysine residues such as LH1 and P4H1 and subsequent excessive glycosylation modifications [11–13]. The presence of both CRTAP and P3H1 proteins in the complex is interdependent, and mutations in either of the CRTAP and LEPRE1 genes result in loss of activity of both proteins; therefore, the phenotypes of OI resulting from mutations in both genes are similar. The third member of the complex, the CypB protein, is encoded by the PPIB gene, and mutations in the PPIB gene result in Sillence type IX OI, which is extremely rare [14]. Defects in CypB proteins have little effect on the activity of CRTAP and P3H1 proteins and mainly affect the folding of collagen chains [15].

Trimeric Intracellular Cation-B (TRIC-B) channels, encoded by the TMEMB38 gene, are widely distributed in the endoplasmic reticulum, regulate the transmembrane flux of K ions, and work in tandem with inositol 1,4,5-trisphosphate receptor (IP3R)-mediated calcium ion release to maintain endoplasmic reticulum membrane electroneutrality. Calcium ions are cofactors for multiple enzymes involved in type I collagen modification and folding, and TRIC-B channel deficiency cause abnormal collagen modification and folding, resulting in Sillence type XIV OI [16–18]. In patients with Sillence type XIV OI, the degree of phenotypic variability is striking, ranging from asymptomatic individuals to severe OI. In addition, patients exhibit a combination of clinical features such as hip inversion, fractures, and long bone curvature [19]. In general, most patients with Sillence type XIV OI exhibit moderate OI severity.

### Type 1D: type I collagen folding and cross-linking defects (Sillence type X and XI, untyped OI)

After the triple helix structure of pre-type I collagen is folded, the heat shock protein (HSP47) encoded by the SERPINH1 gene binds to the triple helix structure as a chaperone protein and maintains its stability, preventing premature collagen fibril formation, and HSP47 can help the folded pre-type I collagen shuttle from the endoplasmic reticulum to the Golgi [20], HSP47 defects result in Sillence type X OI. HSP47 reaching the Golgi needs to dissociate from pre-type I collagen and return to the endoplasmic reticulum, a process that requires the KDEL sequence of the HSP47 protein to bind to the KEDL receptor (KDELR) on the endoplasmic reticulum to be completed. The KEDLR mutation prevents the HSP47 protein from returning to the endoplasmic reticulum and remains bound to pre-type I collagen, disrupting collagen fibril formation. Six recently reported patients with homozygous mutations in the KDELR2 gene were diagnosed with progressive deformation OI, but molecular typing has not been obtained for OI due to mutations in this gene [21].

**Fig. 1:**
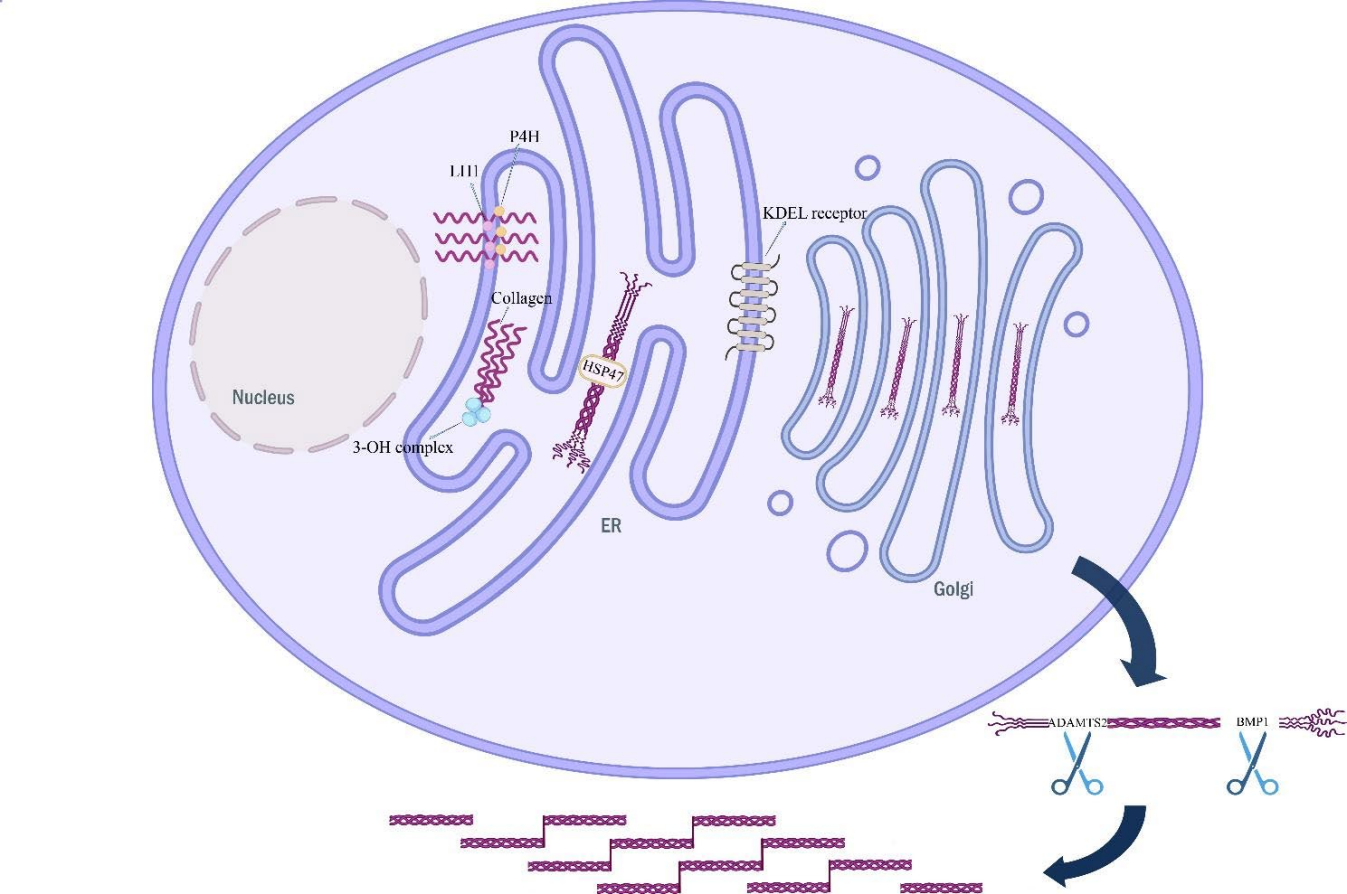
Type I collagen defects and mechanisms 3-OH complex: proline hydroxylase complex; P4H1: prolyl 4-hydroxylase 1; LH1: lysyl hydroxylase 1; HSP47: heat shock protein.

The triple helix formed by the folding of the carboxy- and amino-terminal peptides of pre-type I collagen depends on FKBP65, encoded by the FKBP10 gene, to maintain its structural stability. Lysyl hydroxylase 2 (LH2), encoded by the PLOD2 gene, causes lysine hydroxylation of pre-type I collagen telopeptides, which is required for cross-linking of collagen molecules [22]. Evidence supports a possible interaction between FKBP65 and LH2; patients with FKBP10 mutations have reduced lysine hydroxylation of pre-type I collagen telopeptides and reduced collagen deposition in the extracellular matrix [23, 24]. How FKBP65 affects LH2 activity is unclear. Autosomal stealth mutations occur in FKBP10 leading to Sillence type XI OI, and deletion of LH2 leading mainly to Bruck syndrome, which also causes recessive OI but no typing [25] (Fig. 1).

## Type 2: skeletal mineralization disorders (Sillence type V, VI OI)

Some genetic mutations cause OI not by affecting the type I collagen pathway but by bone mineralization. Sillence type VI OI is caused by an autosomal recessive mutation in the SERPINF1 gene encoding pigment epithelium-derived factor (PEDF), an anti-angiogenic factor that stimulates the expression of osteoprotegerin (OPG). The receptor activator of nuclear factor-κB ligand (RANKL) acts on osteoclast precursor cells by binding to the receptor activator of nuclear factor-κB (RANK) on the surface of osteoclasts. PEDF stimulates the expression of OPG, which inhibits osteoclast maturation by interacting with RANKL and preventing RANKL from binding to RANK.PEDF defects increase osteoclast numbers and bone resorption by affecting the OPG/RANKL/RANK pathway, resulting in reduced bone mineralization [26–28]. Similar to Sillence type III OI, Sillence type VI OI has a severe osteodystrophy phenotype.

**Fig. 2:**
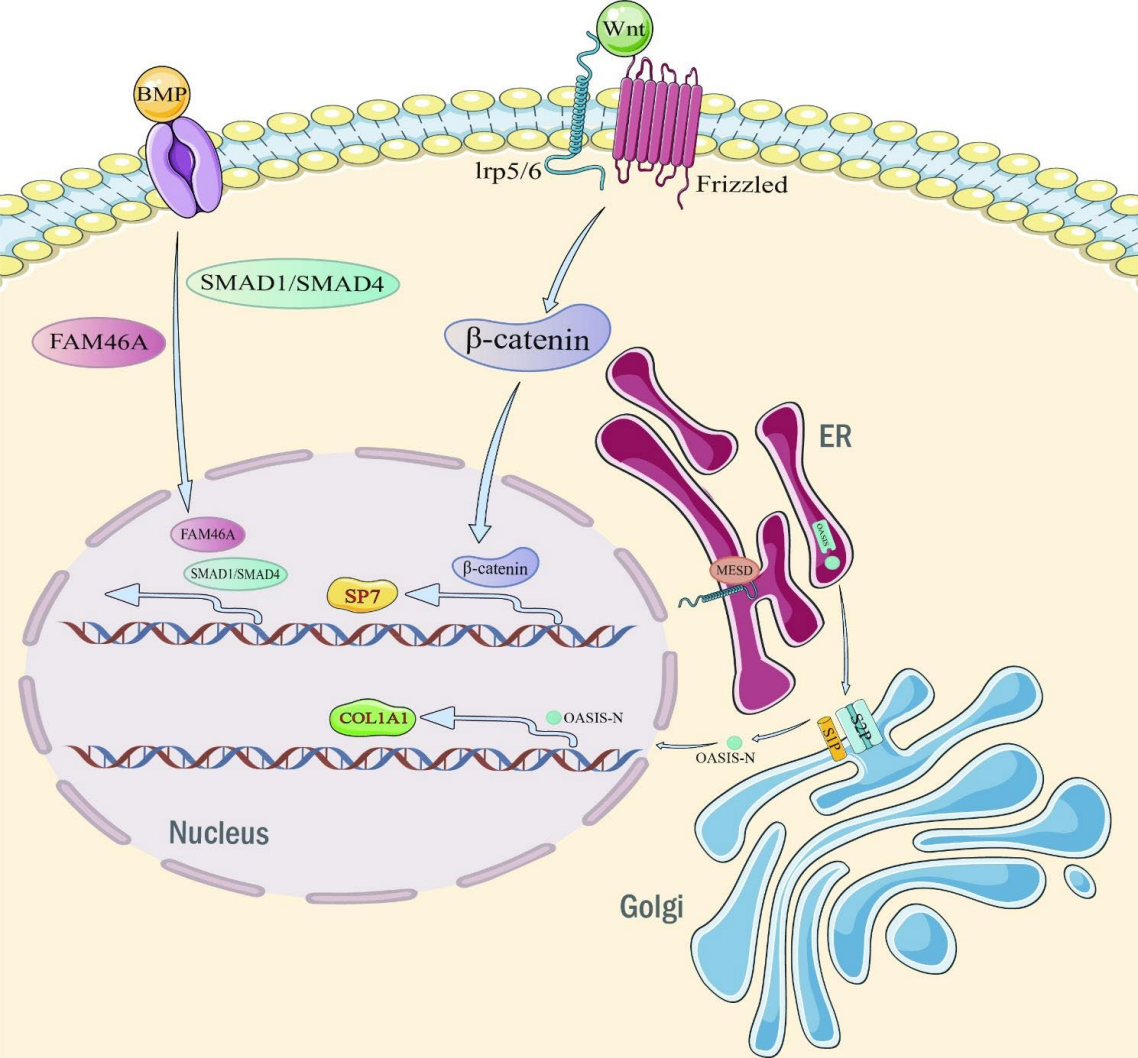
Bone mineralization disorders & osteoblastic differentiation and functional defects OASIS: old astrocyte-specific inducible substance; S1P, S2P: endopeptidases on the Golgi apparatus that together form the Regulatory Intramembrane Protein Hydrolysis System (RIP); WNT1: a secreted ligand; LRP5/6: low-density lipoprotein receptor-related protein 5/6; Frizzled: receptor on the cell membrane, a seventh transmembrane protein; BMP: bone morphogenetic protein.

Sillence type V OI is caused by an autosomal dominant mutation in the IFITM5 gene encoding the bone-restricted interferon-induced transmembrane protein-like protein (BRIL) [29]. BRIL is a transmembrane protein that is enriched during bone mineralization and plays a crucial role in the bone mineralization process. Almost all patients with Sillence type V OI have the same heterozygous mutation in the IFITM5 gene (c.-14 C > T), which occurs in the non-coding region of the IFITM5 gene and results in the addition of 5 amino acids to the amino terminus of the BRIL protein, and this mutation appears to have a function-enhancing effect, leading to increased SERPINF1 expression and PEDF secretion. These patients exhibit an increase in osteoid, crust growth, and calcification of the interosseous membrane of the forearm. Radial head dislocation is also a common finding. It is usually a moderate OI, similar in severity to Sillence type IV OI, in which patients do not have blue sclera or dentin formation insufficiency [30]. Some patients are not suspected to have Sillence type V OI until DNA sequencing. However, the p.S40L (C.119 C > T, p.Ser40Leu) substitution mutation occurring on BRIL causes reduced SERPINF1 expression and PEDF secretion and is the specific mutation causing Sillence type VI OI [31]. Patients possessing this mutation have even more severe bone dysplasia than the typical Sillence type VI OI. The mechanism implied by the interaction of BRIL and PEDF here remains to be further elucidated (Fig. 2).

## Type 3: defective osteoblast differentiation and function (OI types XVI, XVIII, XV, XII, XVII)

In recent years, mutations in genes related to osteoblast differentiation have been shown to be associated with OI. One gene that plays an important role at the osteoblast level is CREB3L1, encoding the old astrocyte specifically induced-substance (OASIS), which is subject to autosomal recessive mutations leading to Sillence type XVI OI. To date, CREB3L1/OASIS defects have been reported in only 5 OI families or individuals. In one Lebanese family, an in-frame deletion of a single-residue codon (P.lys312del) resulted in a mild phenotype (fracture, blue sclera) in heterozygotes, and this mutation caused prenatal/perinatal lethal OI in pure heterozygotes, similar to Sillence type II OI, as a result of mutations in the type I collagen gene [32]. In the presence of endoplasmic reticulum stress, OASIS is translocated from the endoplasmic reticulum to the Golgi membrane and cleaved by the regulated intramembrane proteolysis (RIP) system, releasing the amino-terminal structural domain for transfer to the nucleus for further induction of target gene transcription [33–35]. The RIP system is a highly conserved cellular signaling mechanism consisting of the endopeptidases S1P and S2P on the Golgi apparatus and is associated with growth, differentiation, and endoplasmic reticulum stress responses [36]. Mutations in the MBTPS2 gene encoding S2P cause OASIS to be inactivated by cleavage, resulting in X-linked recessive Sillence type XVIII OI. Moderately severe X-linked recessive OI was reported in two independent lines from Thailand and Germany due to missense mutations in the MBTPS2 gene encoding S2P [37]. WNT1 is a secreted ligand that binds to the low-density lipoprotein receptor-related proteins 5/6 (LRP5/6) and Frizzled receptor on osteoblast precursor cells to stimulate transcription of genes related to osteoblast differentiation via the β-catenin signaling pathway [38]. Heterozygous mutations in the WNT1 gene cause osteoporosis and homozygous mutations cause Sillence type XV OI [39]. Sillence Type XV OI is characterized by short stature, multiple vertebral compression fractures, kyphosis, and severe long bone fractures with phenotypic severity ranging from moderate to progressive deformity. About half of the patients with WNT1-related OI have neurological or brain abnormalities, including dilated ventricles with atrophic changes, cerebellar hypoplasia with short midbrain or type I Chiari malformation, and about 40% have severe intellectual disability or developmental delay [40]. A striking feature is the asymmetry of microcephaly that can be observed in some patients. In addition, all patients with developmental delays or neurological deficits exhibited bilateral ptosis, a unique finding that may contribute to the diagnosis of WNT1-OI [40]. The SP7 gene encodes an osteoblast-specific transcription factor (Osterix) necessary for bone formation and is a target gene of the WNT1 pathway. Mice with deletion of the SP7 gene exhibit insufficient osteoblast differentiation and reduced expression of osteoblast markers, and autosomal recessive mutations in this gene result in Sillence type XII OI [41] (Fig. 2).

## Type 4: unclassified and untyped OI

**Fig. 3:**
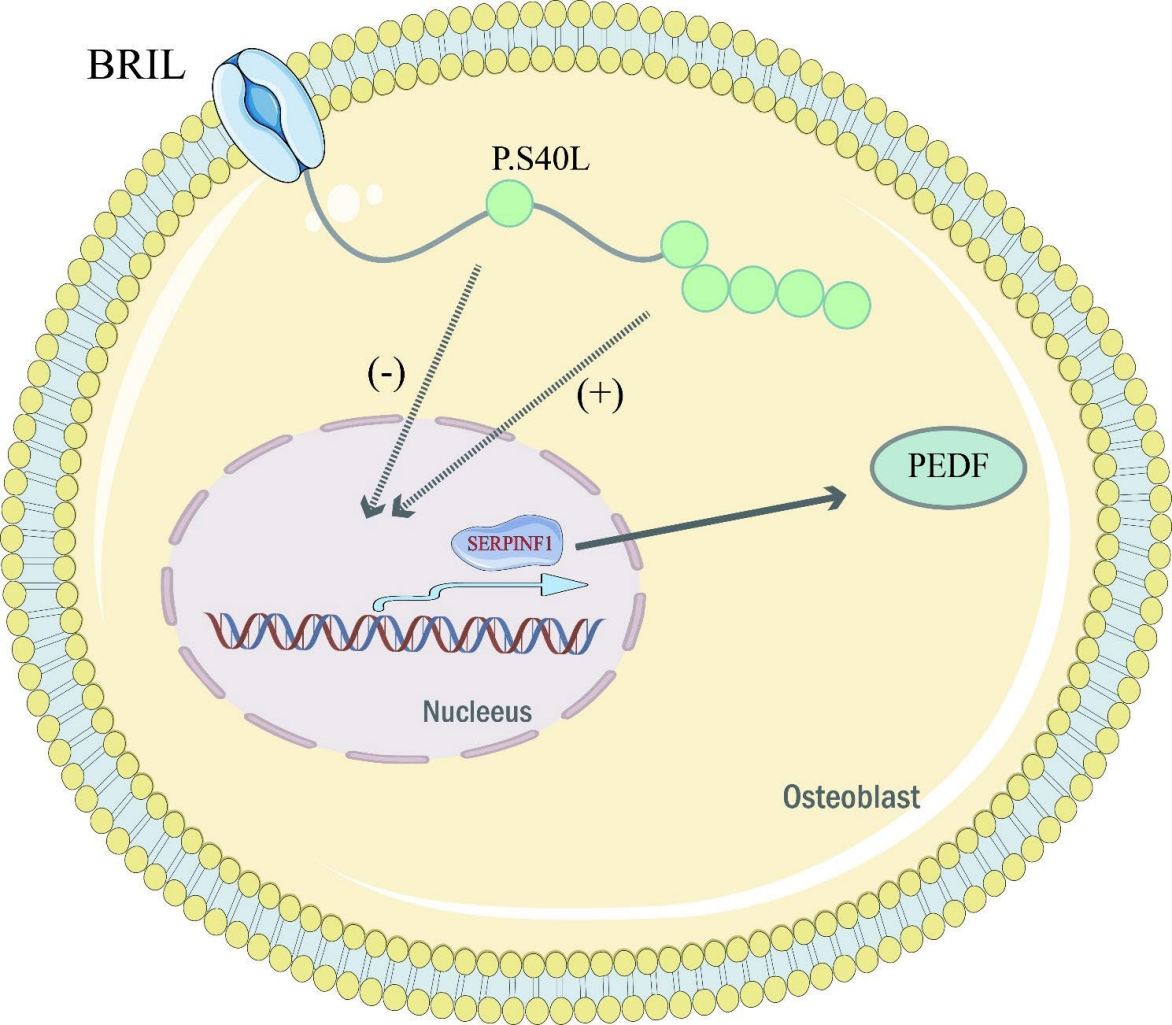
Unclassified and typed OI PEDF: pigment epithelium-derived factor; BRIL: bone-restricted interferon-induced transmembrane protein-like protein.

The FAM46A variant has been reported to be associated with autosomal recessive inheritance of retinitis pigmentosa [5]. However, FAM46A is highly expressed in mouse embryonic skeleton and human osteoblasts, suggesting that it may play an important role in bone development [42]. FAM46A is a regulator in the bone morphogenetic protein (BMP)/transforming growth factor β (TGF-β) signaling pathway, whose function is largely unknown. During Xenopus development, FAM46A induces transcription of BMP target genes by interacting with SMAD1/SMAD4 43. Mice with FAM46A recessive deficiency exhibit a distinct OI phenotype, and FAM46A is thought to be the causative gene for autosomal recessive OI but has not been classified and typed. Interestingly, the FAM46A homozygous mutation was found in children initially thought to have Stuve-Wiedemann syndrome, which reportedly results in a severe autosomal recessive OI with congenital lower limb curvature, fractures, dental abnormalities, and blue sclerae diagnosed in the first year of life [42]. The MESD gene encodes the endoplasmic reticulum chaperone protein of the WNT1 receptor LRP5/6, and mutations in this gene result in autosomal recessive OI [44]. The complex of LRP5/6 and Frizzled acts as a receptor for WNT1 and is an indispensable component of the typical WNT1 signaling pathway. MESD functions in the endoplasmic reticulum, MESD-deficient mice are lethal and show impaired LRP5/6 protein transport, and mutant MESD in MESD-deficient OI patients retains chaperone protein and transport of LRP5, but not in the endoplasmic reticulum [44, 45]. A recent paper reported a compound heterozygous shift mutation in exon 2 and exon 3 of MESD resulting in a stillbirth with multiple intrauterine fractures and severe skeletal malformations [46], suggesting a crucial role for MESD in early skeletal development. However, since the specific mechanism by which MESD causes OI has not been investigated, the classification and typing of OI caused by this gene have not been performed (Fig. 3).

The CCDC134 gene is a newly identified candidate gene for OI pathogenesis, and homozygous mutations in this gene cause autosomal recessive OI. The CCDC134 gene encodes a widely expressed secretory protein involved in the regulatory mechanism of the intracellular mitogen-activated protein kinase (MAPK/ERK) signaling pathway. The CCDC134 mutation results in increased ERK1/2 phosphorylation, decreased OPN mRNA and COL1A1 expression, and reduced osteoblast differentiation by affecting the MAPK/ERK signaling pathway [47, 48]. The mechanism by which mutations in the CCDC134 gene lead to OI may be multifaceted, and no classification and typing of mutations caused by this gene has been performed.

## Conclusion

In recent years, an increasing number of rare genes have been shown to cause OI, and the pathogenic mechanisms of these genes have become a hot topic of current interest and research. The discovery of OI causative genes and the study of causative mechanisms could open up different pharmacological therapeutic perspectives in forms of OI due to alterations of very different metabolic pathways. For example, recent studies of anabolic agents, such as antisclerostin and antitransforming growth factor-beta (anti-TGFβ) antibody, in OI mouse models show improvement in both bone mass and bone strength and may ameliorate the phenotype of OI patients in the future [49].

OI symptom and phenotype variation are related to abnormal interactions of mutant collagen helices with other ECM molecules, rather than to abnormal structure, physical properties or interactions among mutant helices [50]. Furthermore, putative individual genetic variations of other ECM molecules might also modulate the OI outcome. Interestingly, both in vitro and in vivo data from OI animal models indicate that intracellular homeostasis and cytoskeletal organization have a role in modulating OI severity [50]. By integrating the careful clinical tracking of patients, Garibaldi N et al. used the COL1A1 and COL1A2 sequence variant databases with the work of in vitro and in vivo models, thereby paving the way for accurate prognoses and the identification of effective treatment [51].

Classification of OI based on the molecular pathogenesis is the basis for analyzing the genotype and phenotype correlation of patients, which can provide the evidence of classification and guide the accurate clinical diagnosis of OI, predict the severity of the phenotype of OI patients, and assess the prognosis of patients. However, there remain complex conditions that may escape accurate classification. Clinicians should recognize the potential limitation and incorporate other clinical parameters to improve the prognostication and surveillance strategy of OI. With the continuous development of research and science and technology, the molecular classification of OI will be increasingly improved and new breakthroughs in the study of its pathogenic mechanisms will be made.

## Data Availability

Not applicable.

## Abbreviations

AD: Autosomal dominant
ADAMT-2: A disintegrin and metalloproteinase with thrombospondin motifs 2
AR: Autosomal recessive
BMP1: Bone morphogenetic protein 1
BMP: Bone morphogenetic protein
BS: Bruck syndrome
CRTAP: Cartilage-associated protein
CypB: Cyclophilin B
EDS: Ehlers-Danlos syndrome
ERK: Extracellular signal-regulated kinase
HSP47: Heat shock protein
IP3R: Inositol 1,4,5-trisphosphate receptor
KDELR: KEDL receptor
LEPRE1: Leucine proline-enriched proteoglycan (leprecan) 1
LH1: Lysyl hydroxylase 1
LH2: Lysyl hydroxylase 2
LRP5/6: Lipoprotein receptor-related proteins 5/6
MAPK: Mitogen-activated protein kinase
NMD: Nonsense-mediated mRNA decay
OASIS: Old astrocyte specifically induced-substance
OI: Osteogenesis Imperfecta
OPG: Osteoprotegerin
P3H1: Prolyl 3-hydroxylase 1
P4H1: Prolyl 4-hydroxylase 1
PEDF: Pigment epithelium-derived factor
PTC: Premature termination codon
RANK: Receptor activator of nuclear factor-κB
RANKL: Receptor activator of nuclear factor-κB ligand
RIP: Regulated intramembrane proteolysis
SERPINF1: Serpin Family F Member 1
TGF-β: Transforming growth factorβ
TRIC-B: Trimeric Intracellular Cation-B
WNT1: Wnt family member 1
XR: X-linked recessive
